# The effect of fluid shear stress on fibroblasts and stem cells on plane and groove topographies

**DOI:** 10.1080/19336918.2020.1713532

**Published:** 2020-01-16

**Authors:** Xing Lei, Bin Liu, Hao Wu, Xiao Wu, Xiu-Li Wang, Yue Song, Shuai-Shuai Zhang, Jun-Qin Li, Long Bi, Guo-Xian Pei

**Affiliations:** a Department of Orthopedics, Xijing Hospital, The Fourth Military Medical University, Xi’an, China; b Department of Orthopedic Surgery, Linyi People’s Hospital, Linyi, China; c Department of Engineering Mechanics, School of Aerospace Engineering, Tsinghua University, Beijing, China; d State Key Laboratory of Molecular Engineering of Polymers, Department of Macromolecular Science, Fudan University, Shanghai, China

**Keywords:** Frequency, alignment, polarity, adhesion, differentiate, migration

## Abstract

In this study, we aimed to study the effect of fluid shear stress on fibroblasts and BMSCs on plane and groove topographies. The results showed that 0.6-Hz stress had the greatest influence on the alignment, polarity, migration and adhesion of fibroblasts on plane by increasing the expression of reoriented actin and vinculin; whereas 1.0-Hz stress promoted differentiation of fibroblasts into myofibroblasts by increasing Col-I and α-SMA expression. Interestingly, under the given frequency stress, the groove structure strengthened the above characteristics of fibroblasts beyond adhesion, and promoted differentiation of BMSCs into myofibroblasts. The above results indicate that 0.6 Hz may improve the implant-tissue sealing, while 1.0-Hz stress probably causes the disordered fiber deposition around implants.

## Introduction

The introduction of implants such as dentures, steel nails and prosthetic valves has greatly improved the lives of millions of patients worldwide. However, accompanying complications at their interfaces, such as fibrotic encapsulation, infection or avulsion, etc., severely impede the integration between the implants and soft tissue [1,2]. In order to improve the sealing of implants, most researchers have done a lot of work on the surface modification of materials and achieved great results [3,4].

It is generally divided into two types: (i) Chemical modification, i.e. surface binding functional chemical groups [5]; (ii) Physical modification, it refers to altering the surface topography of implant by mechanical, physical or chemical methods, such as grooves, porous, square or cylindrical patterns reported, to optimize the sealing performance [5–7]. Chemical modification is often used to explore the response mechanism of cells to the environment [8–10], while physical modification has achieved good results in clinical application due to its reproducibility, non-degradability and stability [5,11,12].

On the other hand, many implants are usually in the dynamic environment of the human body, such as pacemakers, glucose sensors and external fixation, but few studies pay attention to the influence of these dynamic forces on the material-tissue integration. To solve this issue preliminarily, in this study, we designed a mini-lab oscillator (Fig. S1) to provide a reciprocating fluid shear stress to observe the behavior responses of fibroblasts and stem cells, major participants in tissue healing and fibrosis [13–15].

Firstly, titanium alloy plane, the commonly used surface pattern of implants, was set as a baseline substrate for fibroblasts to investigate cellular response trend to gradient fluid forces. The maximum frequency of the oscillator was set to 1.0 Hz, followed by 0.6 Hz and 0.2 Hz, with the static culture as a control. Then, because groove pattern has a biomimetic texture orientation similar to normal collagen architecture in tissue or organ and better results in promoting interface integration than tapered pits, porous or smooth control surfaces in static state [16–18], we chose the frequency stress that was most sensitive to cell orientation in the previous step to determine its effect on fibroblast behaviors on groove structure, and to observe whether the above advantages of grooves would be enlarged in the flow field. Finally, in order to find out whether frequency could accelerate tissue healing or cause fibrosis through synergistic effect of fibroblasts and stem cells, we exerted the most sensitive frequency stress to the differentiation of fibroblasts into myofibroblasts in the first step to stem cells to investigate the effect of the frequency on stem cell differentiation, because the differentiation into myofibroblasts is a key event in pathophysiological tissue healing [19,20], which can be specifically labeled by Col-I and alpha-smooth muscle actin (α-SMA) [21,22].

## Materials and methods

### Samples preparation

Commercial square TC4 titanium alloy plate (10 mm×10 mm length, 1 mm thick) with smooth surface (Sa = 4.02 ± 0.82 nm) were purchased from Shenzhen Chuangyifu Metal Materials Co., Ltd (Fig. S2A). For groove processing, Xi’an Institute of Optics and Precision Mechanics, Chinese Academy of Sciences, was commissioned to use a commercially available Carbide femtosecond laser system (Light Conversion, Vilnius, Lithuania) to fabricate the structure based on the plane sheets. It delivers 200 fs pulses at 1030-nm wavelength with energy of up to 0.3 mJ and a repetition rate of 50 kHz. This provided structures with 60-μm groove width and ridge of 80 μm, and a groove depth of 10 μm (Fig. S2B).

All samples were cleaned by alternately washing 3 times with 90% (v/v) acetone and 90% (v/v) ethanol in an ultrasonic device for 10 min each time. Then, the sheets were sterilized with 90% ethanol for 4 h and washed with sterile PBS before plating the cells. The topography of samples was imaged by LaB6 scanning electron microscopy (SEM).

### Immunofluorescence

All samples were washed with PBS to remove any unattached cells and fixed in situ with 4% paraformaldehyde for 10 min at room temperature. Then, cells were permeabilized for 5 min with PBS containing 0.1% TritonX-100 and unspecific binding blocked with 2% BSA in PBS for 1 h. To visualize focal adhesions (FAs) or differentiation, cells were treated with the three primary antibodies, including mouse anti-vinculin antibody (Sigma; V9131; 1:300), mouse anti-types I collagen (Col-I) antibody (Abcam; ab6308; 1:200), or mouse anti-α-SMA antibody (Sigma; A5228; 1:200) at 4°C overnight, followed by incubation with goat anti-mouse-FITC (Sigma; F0257; 1:100) at 37°C for 1 h. Subsequently, F-actin was stained with phalloidin-TRITC (Cytoskeleton; PHDR1; 100nM) and the nucleus with DAPI (Thermo; P36931; undiluted).

All specimens were then examined under a ﬂuorescence microscope (Carl Zeiss, Oberkochen, Germany) at low magnification (5× or 10×) or a laser scanning confocal microscopy (Nikon Co., Japan) at high magnification (40×). The fluorescent images were quantitatively analyzed using NIH ImageJ software.

### Morphological analysis

At low magnification (5× or 10×), eight or nine visual fields per group were randomly captured for morphological analysis. Cell orientation was defined as the angle between the long axis of cells and the direction of the shear force; cell shape index (CSI) was based on the ratio of the long axis to the short axis. The inclusion criteria of the above two indicators: (i) Cell outline is clear; (ii) Showing cell polarity. Exclusion criteria: (i) intercellular fusion leads to unclear contour; (ii) more than 2 branches; (iii) obviously curved cells; (iv) binuclear or multinucleated cells. Besides, because of the very small proportions in each group, mononuclear spherical cells were not calculated, either. The number of cells was quantified by counting nuclei. For groove patterns, the number of cells on the groove/ridge was counted by using the principle of different intensity of blue fluorescence on the groove/ridge in DAPI (Thermo; P36931; undiluted) staining. The cell area, fluorescence intensity, and the migration were also measured in this work.

At high magnification (40×), more than five images per specimen were randomly visualized for morphological analysis. FAs were quantified using an anti-vinculin antibody, including fluorescence intensity, FA area and number per cell, and FA area per focal contact point. Differentiation from fibroblasts to myofibroblasts was determined by analyzing fluorescence intensity of Col-I and α-SMA [21,22].

All images were analyzed with NIH ImageJ software.

### Scratch wound assay

NIH-3T3 cells were seeded at a density of 5 × 10^5^/ml onto the plane or groove substrates in 24-well plates and grown to 100% confluence after 8 h of culture. Then, the samples were cultured in serum-free medium. The cell monolayer was scratched with a sterile 200-μl pipette tip and washed with PBS to remove cellular debris. For groove specimens, the scratch direction is parallel or vertical to the groove structure. After 24 h of working with the 0.6-Hz fluid shear force parallel or perpendicular to the scratch, a new scratch was made parallel to the direction of the first one on each substrate as the initial width, and then stained with Calcein-AM (MedChemExpress, Monmouth Junction, USA) with a final concentration of 0.57μM. The changes in cellular migration were recorded with a ﬂuorescence microscope (Carl Zeiss, Oberkochen, Germany) at low magnification (5×). For each image, distances between one side of scratch and the other were quantified using the NIH ImageJ software, and the spacing was normalized to initial width. The assay was repeated four times.

### Protein extraction and western blotting analysis

Total cellular protein was extracted with RIPA lysis buffer, and protein concentration of lysates was measured by BCA Protein Assay Kit (Thermo, Waltham, USA). The protein samples were stored at −80°C for further analysis. Subsequently, equal amounts of proteins were separated by 10% SDS-PAGE gel and then transferred onto polyvinylidene difluoride (PVDF) membranes (Millipore, Billerica, USA). After blocking in 5% non-fat milk in TBST buffer for 1 h at room temperature, the membranes were incubated with three primary antibodies, respectively, including mouse anti-vinculin antibody (Sigma; V9131; 1:1500), mouse anti-Col-I antibody (Abcam; ab88147; 1:2000), mouse anti-α-SMA antibody (Sigma; A5228; 1:2000), or mouse anti-β-actin antibody (Affinity; T0022; 1:3000) at 4°C overnight, followed by incubation with HRP-linked goat anti-mouse IgG (H + L) (Affinity; S0002; 1:3000) at 37°C for 1 h. After washing the membranes with TBST, the band signals were visualized by using Affinity® ECL Reagent. Finally, the membranes were exposed and analyzed by using Luminescent Image Analyzer (Amersham, Uppsala, Sweden). The results of each blot were normalized against β-actin protein expression.

### Scanning electron microscopy (SEM)

After 24 h of fluid force acting on NIH-3T3 cells seeded onto the groove substrates, cell morphologies were assessed via SEM (Tescan, Czech). Brieﬂy, the samples were ﬁxed with 2.5% glutaraldehyde at 4°C for 4 h, dehydrated through a graded series of ethanol, critical-point dried, sputtered with gold, and took photos with SEM.

### Statistical analysis

Statistics were performed using SPSS 21.0 (IBM, Armonk, NY) or GraphPad Prism 7 (GraphPad Software Inc., La Jolla, USA) software. Details of speciﬁc tests, sample numbers, and data were descripted in ﬁgure legends. For a few non-normal data, appropriate variable transformations were used for comparison. Statistical signiﬁcance was considered at p < 0.05.

## Results

### The form and calculation of cyclic fluid shear stress at different frequencies

In order to realistically assess the effect of volume fluctuation in the dish caused by evaporation and subsequent supplementation of the culture medium on flow field velocity in dynamic states, we calculated the maximum and average shear stress provided by the oscillator in a frequency cycle for a film thickness from 2.6 to 3.2 mm. The results were shown in Table S1 and S2, from which we found the data were the same with each other under 0.6 Hz or 1.0-Hz stress and almost no difference for 0.2-Hz force, so the above influencing factors could be ignored as they did not affect the accuracy and repeatability of the study.

We selected 2.9 mm as the representative film thickness to explore the change rule of shear stress with time at different frequencies. The results showed that the shear force performed a sinusoidal wave at each frequency, with a maximum and fastest stress at 1.0 Hz, followed by 0.6 Hz and 0.2 Hz (Fig. S3A). Then, by analyzing the distribution of fluid velocity at different positions on the sinusoidal wave under different frequency stresses, we found that although the flow rate generally decreases with the increase of y altitude, it is greatest at the maximum shear stress (i.e., peaks or troughs), followed by the minimum shear stress (i.e., starting, ending, or middle points of the curve) and intermediate shear stress (i.e., other points on the curve) (Fig. S3B-D).

### Analysis of the NIH-3T3 cell morphological responses to fluid shear stress at different frequencies on plane substrates

**Figure 1.**
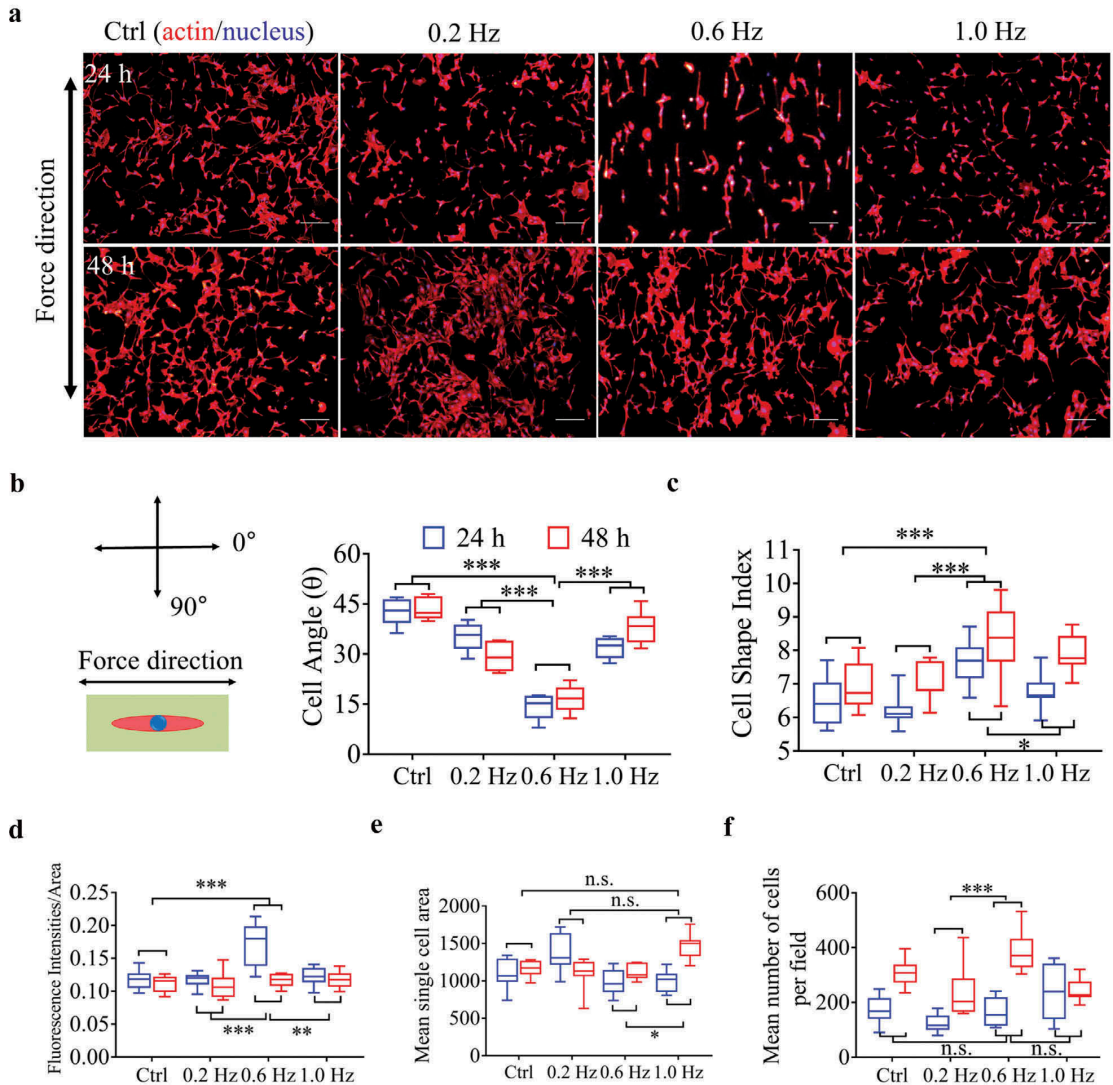
Morphological and quantitative analysis of NIH-3T3 cells on plane for 24 or 48 h. (a) The merged images consist of F-actin (red) and the DAPI-stained nucleus (blue). The force direction is vertical, and static culture as a negative control. Scale bars: 200 μm. (b) The cell direction converted to a range of 0–90°. (c) Box plot of CSI. (d) Average red fluorescence intensity (F-actin). (e) Mean single cell area. (f) Cell counts. Statistical signiﬁcance was assessed by two-way classification ANOVA with Bonferroni’s (b-f) *post hoc* test (n = 9). Mean ± SEM (b, c), Mean ± SD (d-f).

After samples have been placed in the fluid field for 24 and 48 h, we monitored the NIH-3T3 responses to sinusoidal shear stress at different frequencies. Cells cultured in the static state were used as control group. As shown in Figure 1(a,b), S4A and S5, the fibroblasts were well aligned along the direction of 0.6-Hz shear force, followed by 0.2 and 1.0-Hz groups, while the static group presented random orientation. Similarly, the cell polarity (estimated by CSI) was most obvious under 0.6-Hz stress compared with other frequencies (Figure 1(a,c), S4B). Culture time alone also played a positive role in promoting polarity as well (F = 15.638, P = 0.000), which reflected the inherent properties of fibroblasts. Interestingly, when a rank correlation test between angle and CSI on scatter plots was analyzed, we found they were no obvious correlation, no matter in which groups (Fig. S4C), suggesting that they were independent factors and no interaction with each other.

For cell tensions, indicated by the fluorescence intensity of F-actin [23], the 0.6-Hz fluid stress still played the most prominent role in this field (Figure 1(d)), but for cell area, 1.0-Hz group had the greatest spread at 48 h (Figure 1(e), P < 0.0014 vs. others, Bonferroni’s *post hoc* test), although the overall advantage was not obvious (Figure 1(e)). The number of cells increased with the prolongation of incubation time in all groups (F = 60.145, P = 0.000). Especially in 0.6-Hz group, the cell number increased most significantly at 48 h compared with other groups, indicating that 0.06-Hz fluid force stimulated cell proliferation (Figure 1(f), P < 0.029 vs. others, Bonferroni’s *post hoc* test).

### Analysis of the NIH-3T3 cell adhesion and differentiation reactions to fluid shear stress at different frequencies on plane substrates

**Figure 2.**
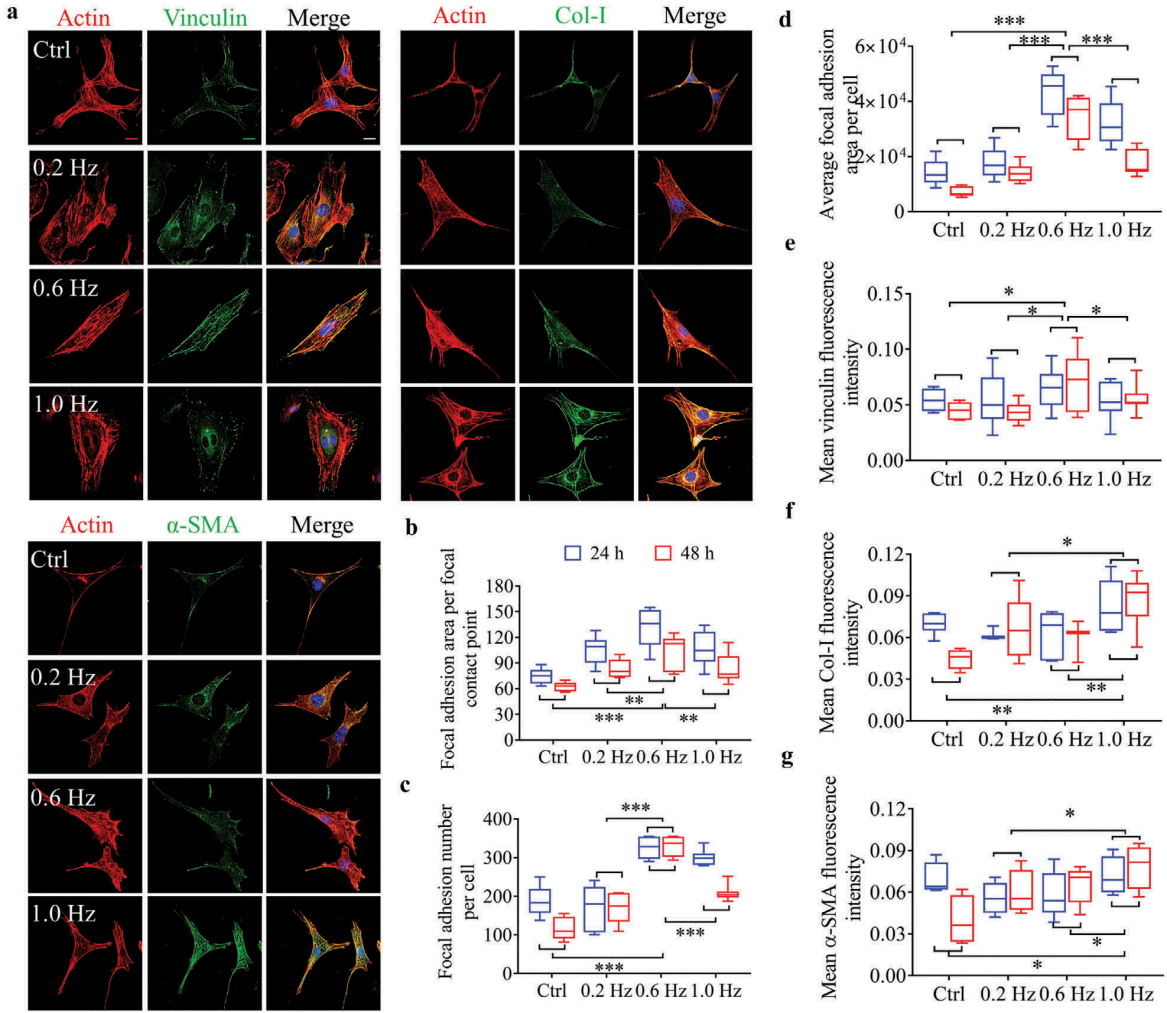
Fluorescence analysis of adhesion and differentiation of NIH-3T3 cells incubated on plane substrates. (a) Images of cell morphology at 24 h under high magnification. Left, Green showing vinculin, Col-I or α-SMA fluorescence. Right, the merged images overlapping of three colors: red (actin), green (vinculin, Col-I or α-SMA) and blue (nucleus). Scale bars = 20 μm. (b) Focal adhesion area per focal contact point. (c) Focal adhesion number per cell. (d) Mean focal adhesion area per cell. (e) Mean vinculin fluorescence intensity. (f) Mean Col-I fluorescence intensity. (g) Mean α-SMA fluorescence intensity. Statistical analyses: two-way classification ANOVA with Bonferroni’s (b-g) *post hoc* test (n ≥ 5, cell counts>20). Mean ± SD (b-g). n.s., no statistical significance; *, P < 0.05; **, P < 0.01; ***, P < 0.001.

To quantitate and compare cell adhesion and differentiation under shear stress of different frequencies, we analyzed three green fluorescent labeled proteins, including vinculin, Col-I and α-SMA (Figure 2(a), S6A). Vinculin is a core component of FA plaques and plays a critical role in cell adhesion [24]. The data showed that the average size of dot-shaped plaques of vinculin first highly raised from the control to the 0.6-Hz group and then decreased at the 1.0-Hz group, showing a peak at the 0.6-Hz group (Figure 2(b)). The vinculin sizes and counts per cell preformed nearly the same trend, exhibiting larger and more quantity of vinculin-containing focal contacts under 0.6-Hz force (Figure 2(c,d)), suggesting that 0.6-Hz shear force had a more positive impact on cell adhesions, which were verified by the results from vinculin fluorescence intensity (Figure 2(e)) and protein expression (Fig. S6B, C).

With respect to the Col-I and α-SMA, both fluorescence intensity (Figure 2(f,g)) and protein expression (Fig. S6B, C) of them were most sensitive to 1.0-Hz stress. Col-I and α-SMA, as markers of fibroblast differentiation into myofibroblasts, are often used to assess cell differentiation potential [21,22]. Thus, 1.0-Hz sinusoidal stress had the most influences on differentiation, which may also explain the increase of cell area in this group (Figure 1(e)), because myofibroblasts have larger spreading areas than fibroblasts [25,26].

To further verify the above conclusions from statistics, we used multiple stepwise regressions to compare the correlation between frequency, time, and dependent variables. The analysis showed that most parameters in NIH-3T3 cells, including cell alignment, polarity, and adhesion, were more correlated with frequency than time after excluding the 1.0-Hz grouping factor. On the other hand, if all groups were included in the analysis, the most correlation to frequency was an expression of α-SMA and Col-I (see details in Fig. S7, S8), the specific markers of myofibroblast differentiation [21,22]. Therefore, combined with the experimental results and statistical analysis, we prove that alignment, polarity, and adhesion of fibroblasts are most sensitive to 0.6-Hz stress, and differentiation is to 1.0 Hz.

### Analysis of the NIH-3T3 cell morphological responses to 0.6-Hz fluid shear stress on groove patterns

**Figure 3.**
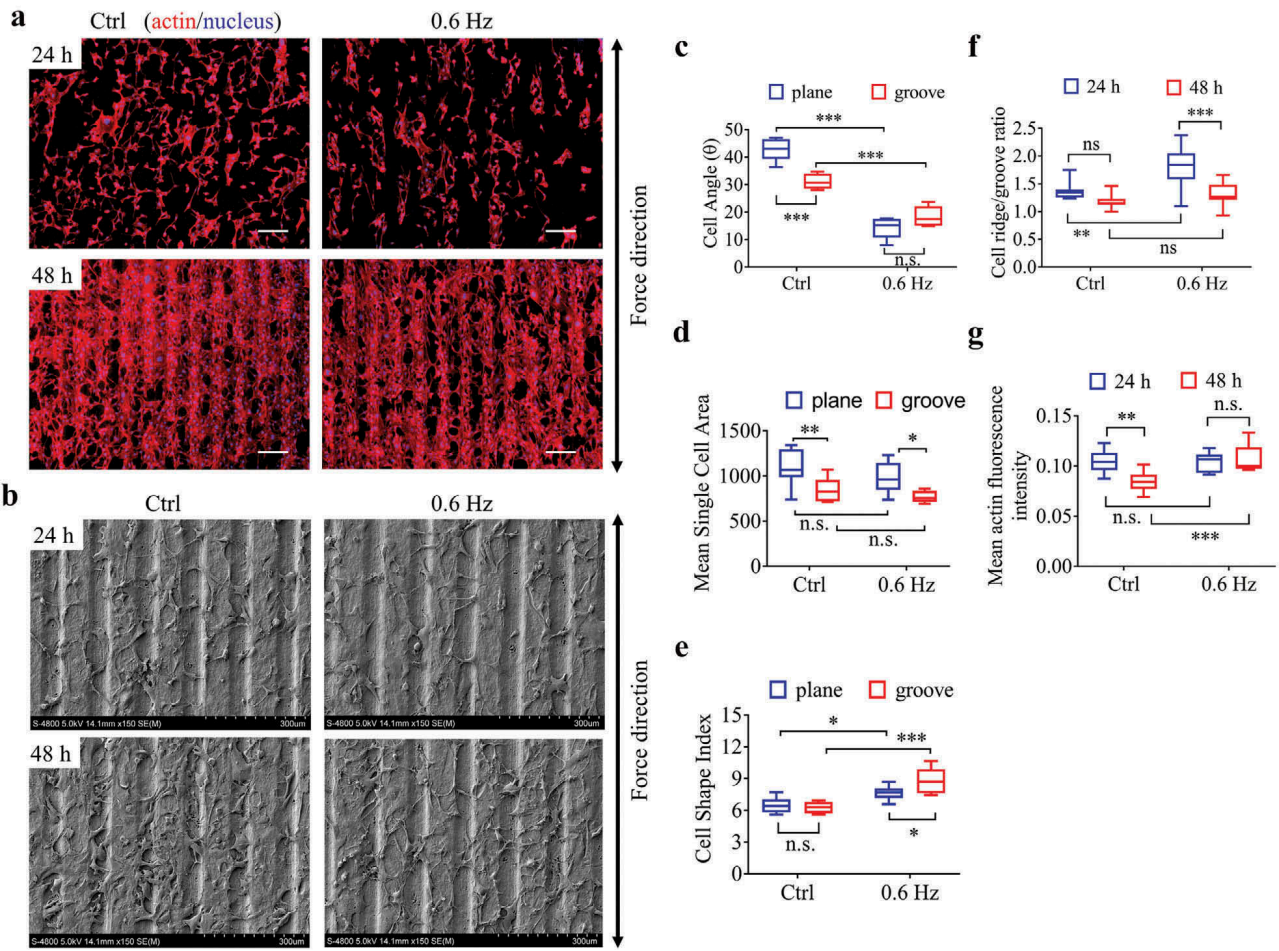
The morphology and quantification of NIH-3T3 cells implanted on groove structures for 24 h and 48 h. (a) The merged images consist of F-actin (red) and the DAPI-stained nucleus (blue). The force direction is vertical, with the static culture as a control. Scale bars: 200 μm. (b) SEM micrographs of fibroblasts at 24 h and 48 h on groove substrates. (c) Cell angle at 24 h. (d) Mean single cell area at 24 h. (e) Cell shape index at 24 h. (f) Ratio of cells on the ridge to groove. (g) Mean F-actin fluorescence intensity of cells seeded on grooves. Statistical analyses: two-way classification ANOVA with Tukey’s (c-g) *post hoc* test (n = 9, cell counts>600). Mean ± SEM (c-d), Mean ± SD (e-g). n.s., no statistical significance; *, P < 0.05; **, P < 0.01; ***, P < 0.001.

The groove structures (groove/ridge width of 60 μm and 80 μm, respectively) with a groove depth of 10 μm were fabricated (Fig. S2B), on which NIH-3T3 cells were seeded, and its behavioral responses to 0.6-Hz fluid force were examined. The results showed that the groove pattern increased the alignment of fibroblasts (Figure 3(a–c)) and reduced the cell area (Figure 3(d)) after 24 h of static culture compared with the plane group, but had no significant effect on cell polarization (Figure 3(e)). The 0.6-Hz shear force not only further enhanced the alignment of fibroblasts on the grooves but contributed to the increase of cell polarity (Figure 3(e)). For the data at 48 h, no analysis was performed because most cells were overlapping with each other and the boundaries were unclear.

After 24 h of static culture, the number of fibroblasts on the ridge was greater than that in the groove, which means the cells were ridge-loving. It is worth noting that 0.6-Hz fluid forces enlarged this advantage and made the ratio even higher. With the prolongation of culture time (48 h), however, this superiority gradually disappeared with the increase of cell number (Figure 3(f)). In terms of the fluorescence intensity of actin, it seemed that the longer the static incubation time, the weaker the intensity on the groove samples, but the result was reversed by the 0.6-Hz dynamic force at 48 h (Figure 3(g)), suggesting the fluid stress could increase cell tensions on the groove substrates.

### Analysis of the NIH-3T3 cell migration and adhesion reactions to 0.6-Hz fluid stress on groove substrates for 24 h

**Figure 4.**
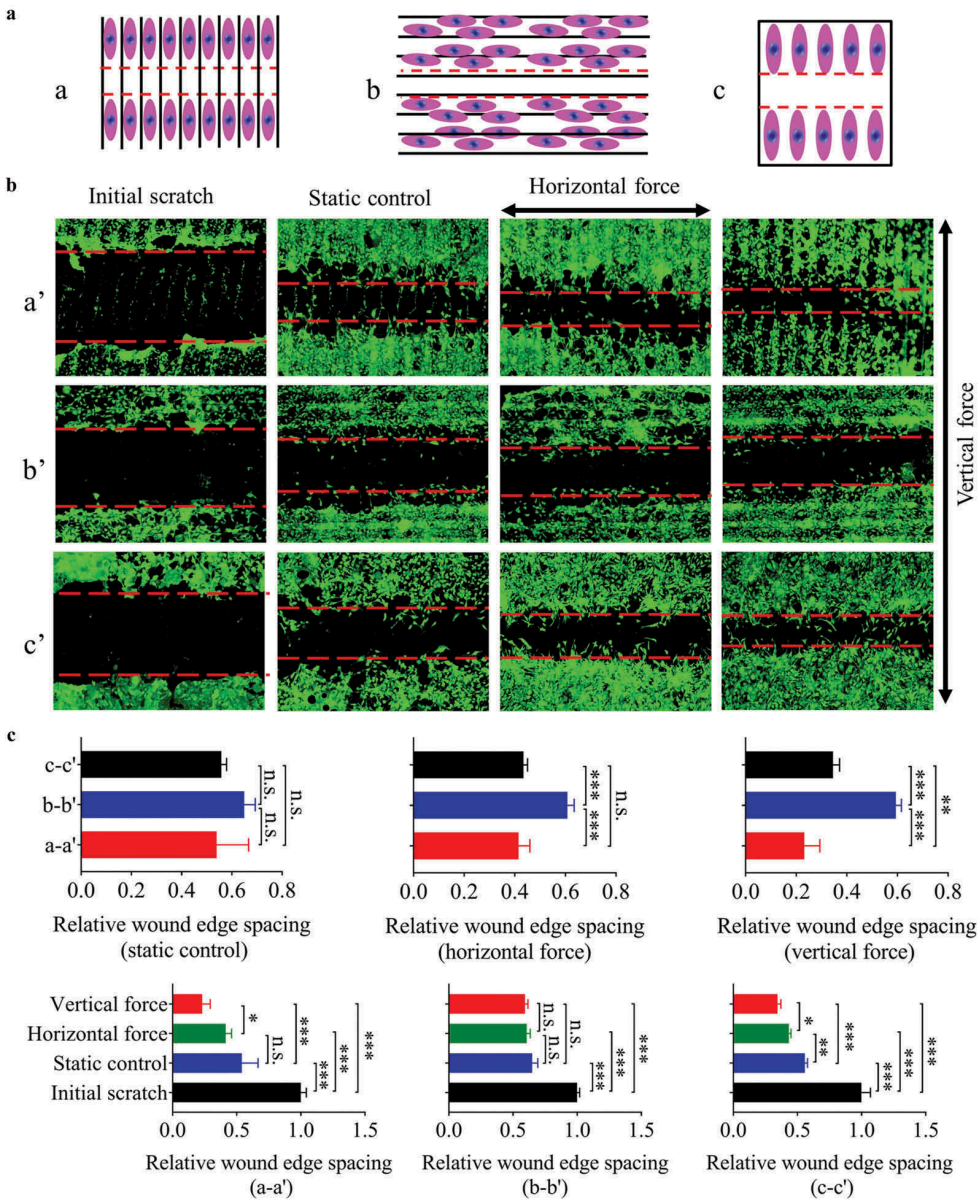
The migration of NIH-3T3 cells seeded on plane/groove substrates for 24 h. (a) The schematic diagram shows the relative position between scratch and groove orientation. a, scratch perpendicular to the groove direction; b, scratch along the groove direction; c, scratch on the plane after the cells are covered. (b) Images of cell migration under low magnification (5×), a’, b’, and c’ correspond to a, b, and c in A, respectively. C, Quantification of the wound widths. Statistical analyses: one-way ANOVA with Tukey’s multiple comparison test (n = 4). Mean ± SEM. n.s., no statistical significance; *, P < 0.05; **, P < 0.01; ***, P < 0.001.

In order to reveal the effect of fluid force on the migration of fibroblasts seeded on the groove pattern, a sketch was designed (Figure 4(a)). The results showed that the different direction combinations of groove and 0.6-Hz fluid force caused different migration velocities for 24 h (Figure 4(b)). In static culture, the substrate structure had no effect on cell migration. Under dynamic state, compared with the plane, the fluid force along the groove direction accelerated the cell motility (Figure 4(c)). The above results are consistent with the previous findings on cell polarity (Figure 3(e)), an indicator of cell migration [25,27]. On the contrary, the force perpendicular to the groove hindered cell migration (Figure 4(c)), possibly due to the blocking effect of the ridges.

**Figure 5.**
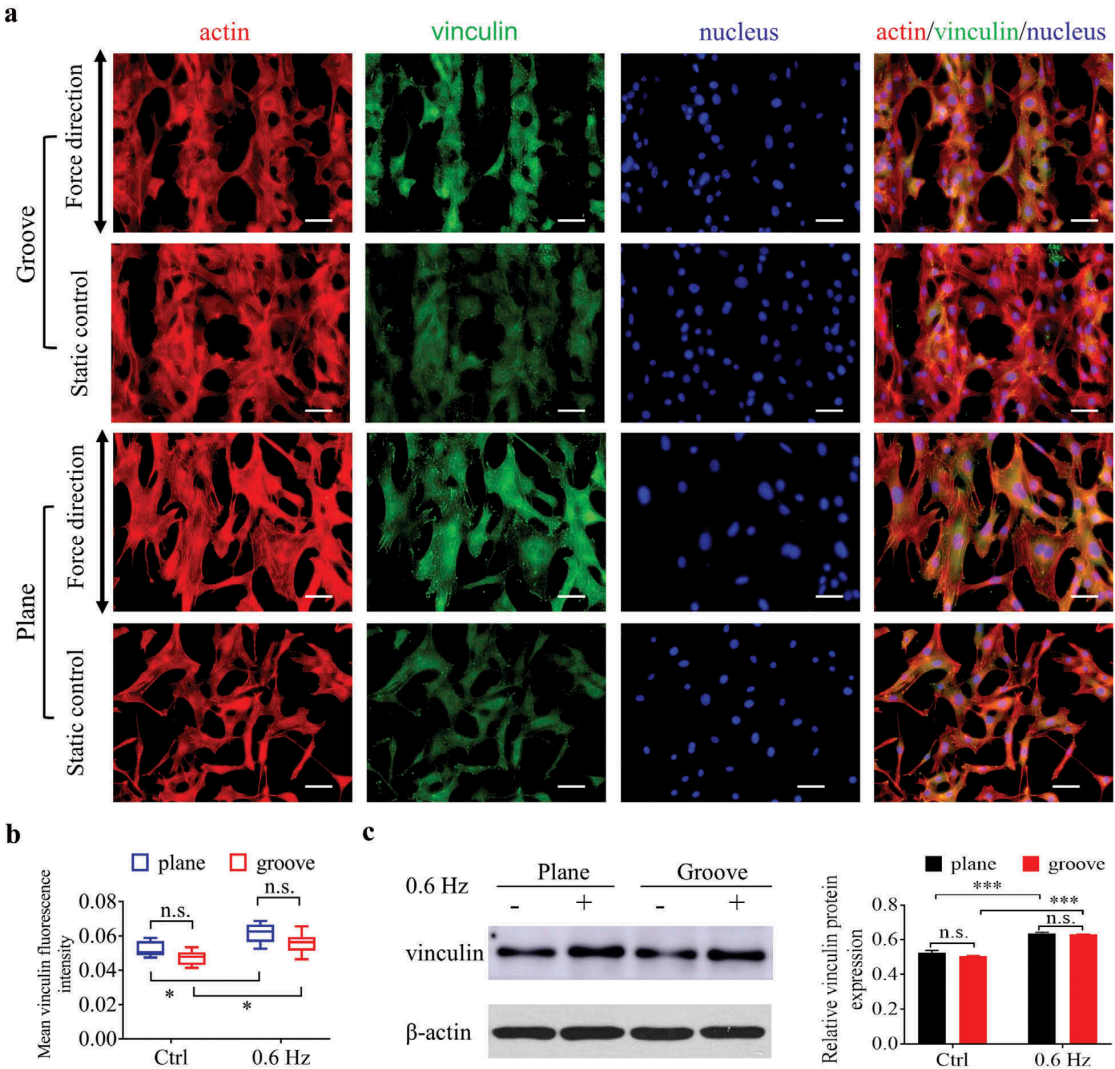
Adhesion of NIH-3T3 cells at 24 h after incubating on groove patterns. (a) Fluorescent images of actin and vinculin of fibroblasts. The merged images consist of F-actin (red), vinculin (green) and nucleus (blue). The force direction is vertical, and the static culture as a control. Scale bars: 100 μm. (b) Quantification of vinculin fluorescence intensity of fibroblasts seeded on grooves. (c) The protein expression (left) and quantitative analysis (right) of vinculin. The values are normalized to β-actin. Statistical analyses: two-way classification ANOVA with Tukey’s multiple comparison test (n ≥ 5, cell counts>50). Mean ± SD. n.s., no statistical significance; *, P < 0.05; ***, P < 0.001.

To investigate the role of 0.6-Hz force in the adhesion of fibroblasts on groove patterns, immunofluorescence and western blotting were used to determine the expression of vinculin after cells were placed in the fluid field for 24 h (Figure 5(a)). As shown in Figure 5, the shear force of 0.6 Hz not only increased the adhesion of cells on the surface of plane, which has been shown previously in this study, it also had the same effect on the cells on the grooves. In addition, the adhesion of fibroblasts had no significant correlation with substrate structure, neither groove nor plane (Figure 5(b,c)).

### Analysis of the BMSCs differentiation response to 1.0-Hz sinusoidal stress on plane and groove samples

**Figure 6.**
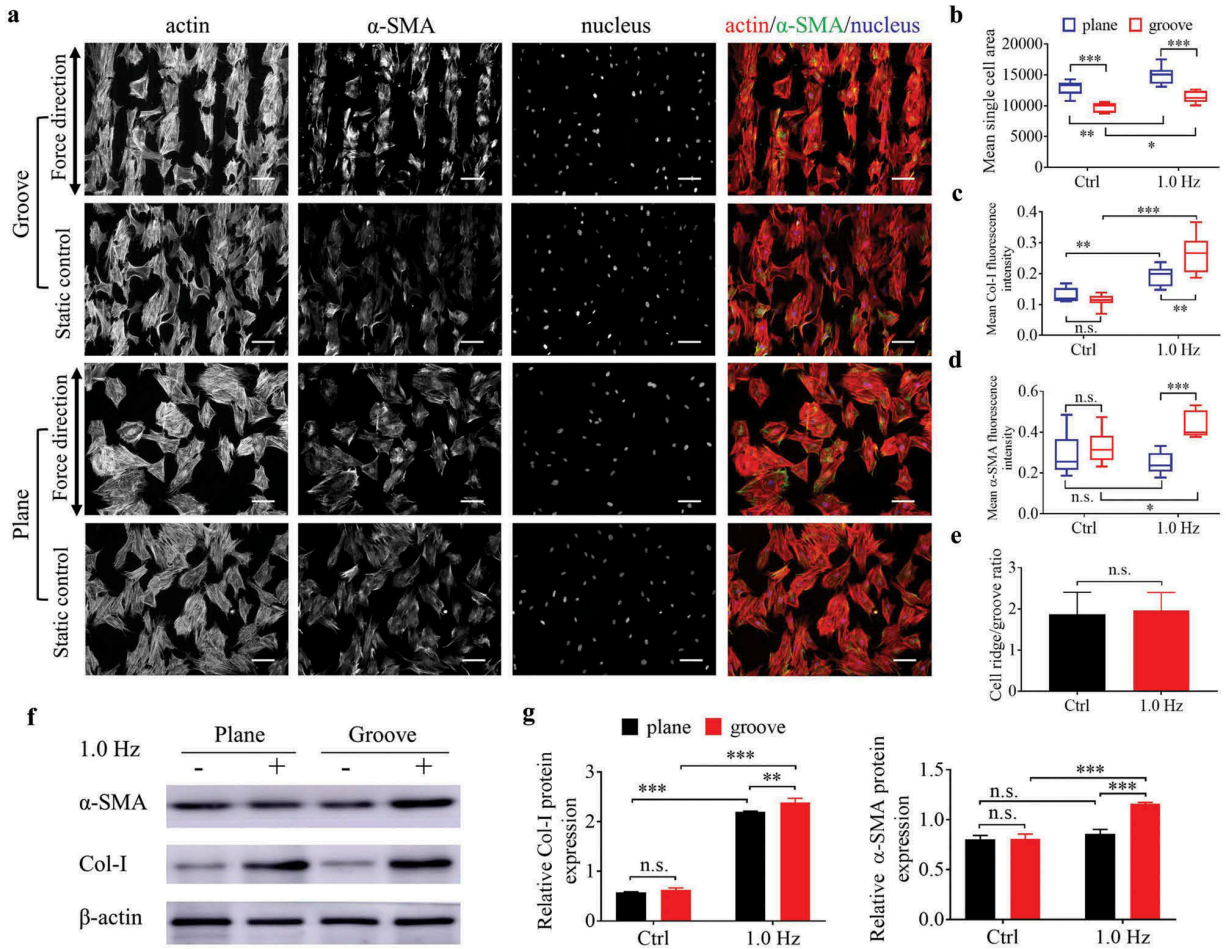
The differentiation of BMSCs seeded on plane and groove structures for 24 h. (a), Fluorescent images of cytoskeleton and α-SMA of BMSCs. Images are taken in black-and-white mode of fluorescence microscopy, and merged pictures are added with pseudo-color, including red (F-actin), green (α-SMA) and blue (nucleus). The force direction is vertical, with the static culture as a control. Scale bars: 100 μm. (b) Mean single cell area. (c–d) Mean Col-I (c) and α-SMA (d) fluorescence intensity. (e) Ratio of cells on the ridge to groove. (f) The protein expression of Col-I and α-SMA. (g) Quantifying the gray value of protein bands, the proteins are normalized to β-actin. Statistical analyses: two-way classification ANOVA with Tukey’s multiple comparison test (b-d, g); two-tailed Student’s t-test (e). n = 9, cell counts>50 (b-e), n = 3 (g). Mean ± SD (b-d, e, g). n.s., no statistical significance; *, P < 0.05; **, P < 0.01; ***, P < 0.001.

To examine whether 1.0-Hz fluid stress can promote the differentiation of stem cells into myofibroblasts, we used mouse BMSCs for fluorescence staining and western blotting to observe the cell response to the dynamic force in the flow field. After 24 h of static culture, the majority of BMSCs on plane was polygon-shaped and showed no obvious polarity compared with fibroblasts (Figure 6(a), S9). Perhaps due to the limitation of ridge width on cell expansion, the average cell area on the groove surfaces was smaller but more spindle-shaped appearance than that on the planes (Figure 6(b)), which is closer to the morphology of myofibroblasts [25,26]. However, there was no difference in the fluorescence intensity of Col-I and α-SMA of stem cells on both types of substrates under static state (Figure 6(c,d), S9).

After adding 1.0-Hz fluid force for 24 h, the parameters reflecting differentiation into myofibroblasts such as cell area, fluorescence intensity and protein expression of α-SMA and Col-I increased (except for the α-SMA on the plane) (Figure 6(c,d,f,g)), suggesting that 1.0-Hz sinusoidal force promoted the differentiation of BMSCs into myofibroblasts on the grooves and increased the collagen deposition on plane. As for the ratio of cells on the ridge to groove, BMSCs preferred to settle in ridges rather than grooves (the ratio>1), similar to fibroblasts, but the force of 1.0 Hz did not enhance this value (Figure 6(e)), possibly due to the spreading-shape of stem cells. The above results suggested that BMSCs had a synergistic effect with fibroblasts in collagen synthesis and wound contraction.

## Discussion

In this work, we firstly found that many parameters of NIH-3T3 cells on plane, including direction, polarity and adhesion, were biphasic-dependent with the increase of frequency stress, with the peak at 0.6 Hz, but the differentiation (into myofibroblasts) was most correlated with 1.0-Hz stress. In other words, there was a threshold that determined the fate of appearance and adhesion of fibroblasts under fluid stress. Below the threshold, the cells were always aligned parallel to the force direction, and polarized obviously and FAs grew with the increase of fluid frequency; above the threshold, however, the cells tended to reorient themselves away from the force direction, and the FAs depolymerize [23,28]. These discoveries implied there is a characteristic frequency between 0.6 Hz and 1.0 Hz, beyond which the alignment, polarity and adhesion of cells would regress, as well as the expression of actin.

Next, we investigated the fibroblasts responses to 0.6-Hz fluid stress on the groove pattern, with the plane as a control. In static culture, NIH-3T3 cells on grooves were better aligned than those on plane due to its ‘contact guidance’ [29]. The narrower the ridge, the better the alignment of cells within a specific dimensional range [30]. The polarization, however, was not affected by the groove structure, which is in line with the results of Kim et al. [16]. Interestingly, after fibroblasts were placed in the 0.6-Hz flow field, both of them got enhanced and became more obvious, as well as the expression of vinculin and actin. Vinculin, as a core element for sensing extracellular mechanical stimuli, coordinates the force-induced cell polarization by linking actin [24,31]. Thus, cell polarization required increased expression of vinculin and actin. Besides, cell polarity is a critical positive indicator of cell migration [25,27], so 0.6-Hz fluid force accelerated the migration of fibroblasts on the groove, without being blocked by the ridge, of course. Furthermore, the direction of cells and speed of migration are mainly maintained by increased actin [28,31], which also enhances the tension and stiffness of the cells, and strengthens the adhesion of the cells with the increased vinculin together [23,24]. These previous findings well explained our findings and the causal relationship between the above parameters. It should be noted that the groove structure strengthened the above characteristics of fibroblasts beyond adhesion under the fluid stress.

**Figure 7.**
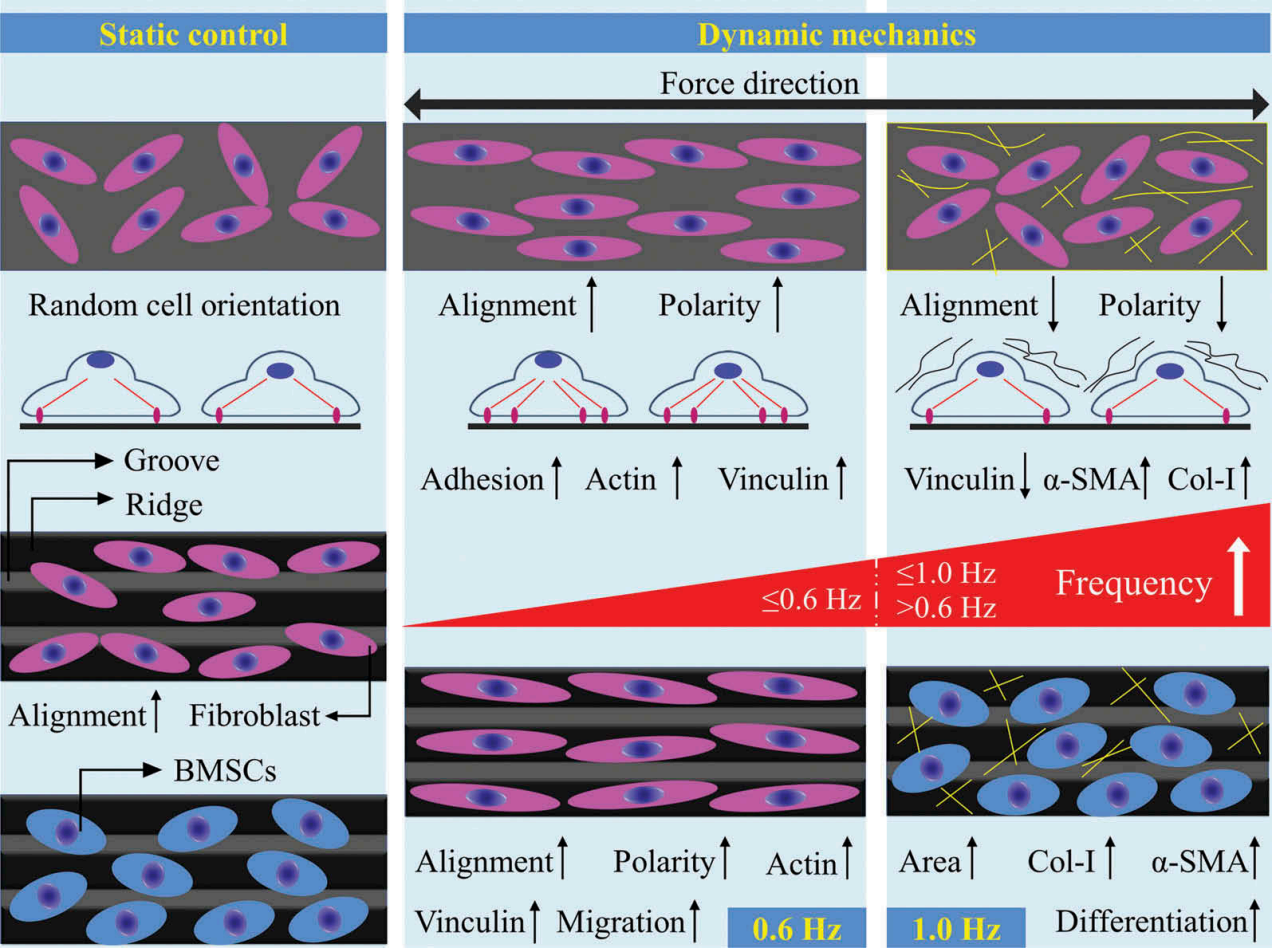
A schematic diagram of the effect of fluid shear stress on the behavior of fibroblasts and BMSCs on plane and groove substrates.

In summary, 0.6-Hz fluid stress affects the many behaviors of fibroblasts, including orientation, polarity, migration and adhesion, by redirecting actin filaments and increasing the expression of actin and vinculin on plane and groove (Figure 7). Well alignment and rapid migration of fibroblasts promote the orderly deposition of collagen and form good tissue architecture, and these characteristics are particularly prominent on groove structure. Besides, increased adhesion improves the firmness between tissue and implants under 0.6-Hz stress. Therefore, 0.6-Hz stress might improve the implant-tissue integration, and groove structure can be a suitable candidate for surface modification patterns of implants.

Differentiation into myofibroblasts is a key event in physiological wound healing and pathological fibrogenesis [19,20], and is characterized by the production of α-SMA and Col-I [21,22]. Here, we found that 1.0-Hz fluid force activated the differentiation of fibroblasts into myofibroblasts and further promoted collagen aggregation and fiber contraction endowed by α-SMA [1]. Unfortunately, we believe this aggregation is relatively disordered and loose due to the inconsistency of cell orientation and FAs depolymerization. At the same time, Col-I produced by BMSCs aggravates the unstructured deposition of collagen and leads to the formation of fiber encapsulation around implants eventually, which was not beneficial to the improvement of implant-tissue integration and organ function. Furthermore, groove structure promoted the differentiation of stem cells into myofibroblasts by increasing the expression of α-SMA and Col-I, suggesting that the pattern further enhanced the pro-fibrotic effect of 1.0-Hz fluid shear stress (Figure 7). Taken together, our works provide new insights into the role of mechanical factors in cell–material interaction and the design of implant surface modification in the future.

## Conclusions

The groove morphology of the implant surface combined with the reciprocating vibration of 0.6 Hz may be an effective strategy to improve material-tissue integration. However, if the applied frequency parameters are increased, such as 1.0 Hz, disordered and thick fibrous capsules may be formed around the implants.

## Data Availability

All data that support the conclusions are available from the authors on request. https://mail.126.com/js6/main.jsp?sid=LBdPdGzzEPKdpnkPjHzzvGfpEEJLjYwQ&df=mail163_letter#module=welcome.WelcomeModule%7C%7B%7D
