# Supplementary material for: The effect of fluid shear stress on fibroblasts and stem cells on plane and groove topographies
Source: Cell Adh Migr. 2020 Jan 16;14(1):12–23. doi: 10.1080/19336918.2020.1713532 (PMC6973306; doi:10.1080/19336918.2020.1713532)
Supplement: Supplemental Material [file kcam-14-01-1713532-s001.docx]

**SUPPORTING INFORMATION**

**The effect of fluid shear stress on fibroblasts and stem cells on plane and groove topographies**

Xing Lei^†‡#^, Bin Liu^†#^, Hao Wu^†^, Xiao Wu^§^, Xiu-Li Wang^¶^, Yue Song^†^, Shuai-Shuai Zhang^†^, Jun-Qin Li^†^, Long Bi^†^, Guo-Xian Pei^†*^

†. Department of Orthopedics, Xijing Hospital, The Fourth Military Medical University, Xi’an, 710032, China

‡. Department of Orthopedic Surgery, Linyi People’s Hospital, Linyi 276000, China

§. Department of Engineering Mechanics, School of Aerospace Engineering, Tsinghua University, Beijing 100086, China

¶. State Key Laboratory of Molecular Engineering of Polymers, Department of Macromolecular Science, Fudan University, Shanghai 200438, China

# Authors contributed equally

*correspondence: Guo-Xian Pei

Department of Orthopedics, Xijing hospital, The Fourth Military Medical University, 15 changle West street, Xi’an 710032, china

Tel +86 29 8477 3524

nfperry@163.com

**
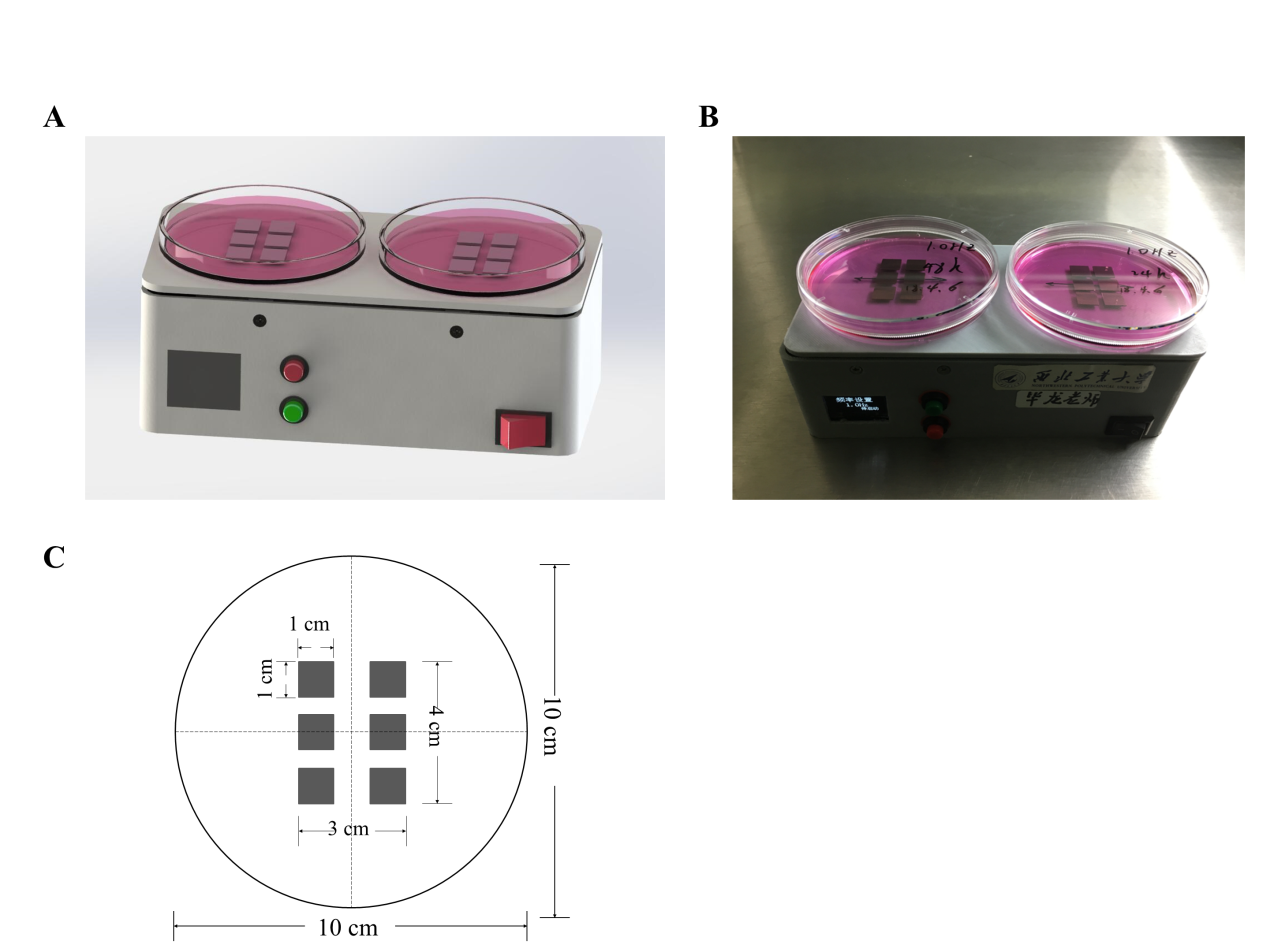
**

**Figure S1. Mini lab oscillator.** A, Oscillator schematic. B, Oscillator physical map. C, Diagram of position arrangement of six plane sheets in a φ100mm cell culture dishes.

**Supplemental materials and methods**

**Cell Culture**

NIH-3T3 (mouse embryonic fibroblast cell line, Cell Bank of the Chinese Academy of Sciences, Shanghai, China) and mouse bone marrow-derived mesenchymal stem cells cells (BMSCs, which were isolated from the femur bone marrow of 2-week-old mice. See our previous published research for more details ^[^[^1^](#_ENREF_1)^]^) were cultured in Dulbecco’s modified Eagle’s medium (DMEM, Hyclone, USA) containing 4.5 g/L d-glucose and supplemented with 10% fetal bovine serum (FBS, Sigma-Aldrich, St. Louis, USA), and 1% v/v penicillin/streptomycin (Sigma-Aldrich, St. Louis, USA) at 37 °C in a humidified atmosphere containing 5% CO_2_. NIH-3T3 or BMSCs cells were seeded at a density of 2×10^4^/ml onto the plane or groove substrates and incubated for 8 h (20% confluence) in 24-well microplates. Cellular analyses were carried out at 24 or 48 h after samples were transferred from microplates to φ100mm cell culture dishes.

**Construction of fluid shear stress models at different frequencies**

To determine the effect of dynamic mechanics on material-tissue integration, we designed and entrusted Northwestern Polytechnical University to make a mini lab oscillator which can provide reciprocating fluid shear force to act on cells (Fig. S1A, B). Two φ100mm cell culture dishes were placed in parallel on the oscillator, with six plane sheets implanted in each dish (Fig. S1C). The oscillator is rechargeable and can be placed in a cell culture incubator to provide power without affecting the cell culture environment. The frequency can be adjusted from 0.2 to 1.2 Hz, and the amplitude is 2 cm. The oscillator worked 18 h per 24 h. Three grades of 0.2, 0.6 and 1.0 Hz were selected as the experimental groups; cells cultured in static state were used as the control group.

We set up 30 ml of culture medium in each dish, but, actually, the volume of liquid was measured to fluctuate in the range of 28~32 ml due to evaporation and later replenishment, so the distance from the liquid surface to the titanium alloy plane, called as film thickness, floated around 2.9 mm (2.6~3.2 mm) in static state [πr^2^×(Film thickness+titanium alloy thickness)=volume of (medium+titanium alloy), i.e. 3.14×5 cm^2^×(Film thickness+0.1 cm)=30 ml+0.6cm^3^ (ml), Film thickness=2.9 mm]. Because the six plane sheets were placed symmetrically on both sides of the midline in a dish, film thickness above the two row specimen surfaces was equal in a frequency cycle. Since the film thickness was very small compared to the diameter of dish (2.9 mm vs. 100 mm), the case could be transferred into one-dimensional condition where the fluid velocity at y point only related to time and the vertical distance between y point and the material surface. Y point means any position above the samples in the fluid. Thus

$u=u\left(y\right)$, (1)

Where *u* is the velocity, and *y* is the vertical distance from a point in fluid to the surface of plane. Then the N-S equation can be converted into

$\frac{\partial u}{\partial t}=\upsilon\frac{\partial^{2}u}{\partial y^{2}}$, (2)

Where *t* is time，*υ* = 1.006×10^-6^ is kinematic viscosity.

The boundary condition is,

$\left.u\right|_{y=0}=u_{wall}$, (3)

$\left.\frac{\partial u}{\partial y}\right|_{y=\delta}=0$, (4)

Where *u_wall_* is the velocity of the plane samples, and *δ* is the film thickness.

That can be solved by numerical method, where FTCS (Forward difference Time, Central difference Space) was used and the node of mesh was 100. The solutions were computed by MATLAB (Mathwork, Inc., USA). We could get shear stress by

$Shear stress= \mu\left. \frac{\partial u}{\partial y} \right|_{y=0}$ (5)

Where μ = 1.005 ×10^-6^ Pa•s is the fluid viscosity.


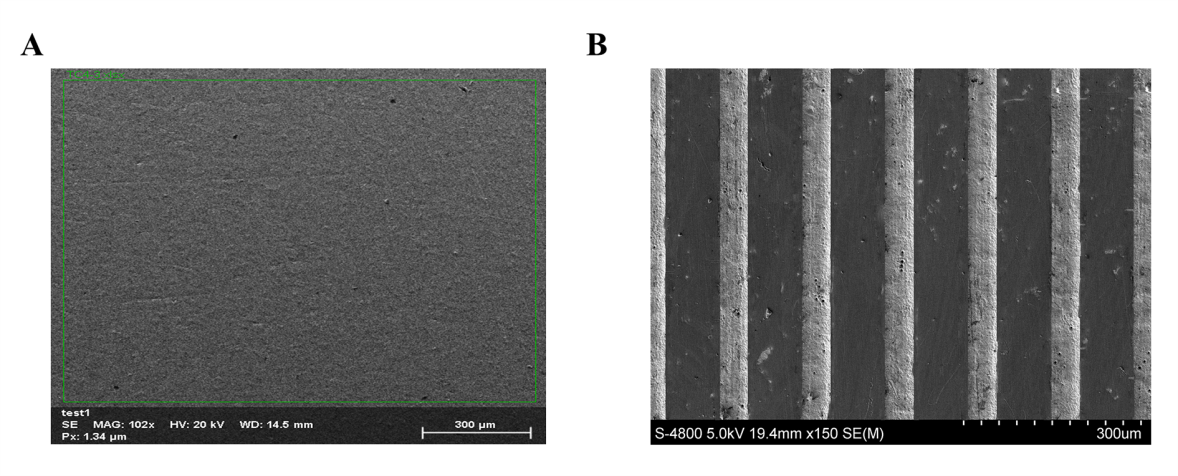


**Figure S2. The surface images of plane/groove substrates were visualized by SEM.** A, plane. B, groove. Groove and ridge width are 60 and 80 μm, respectively. The groove depth was designed to be 10 μm.

**Table S1. Maximum shear stress provided by oscillator in a sinusoidal cycle (dyne/cm^2^)**

| **Film thickness (mm)** | **0.2 Hz** | **0.6 Hz** | **1.0 Hz** |
| --- | --- | --- | --- |
|  | $\begin{matrix} u_{wall}=0.8\pi\sin\left(0.4\pi t\right)\text{ } \\ \text{ (cm/s)} \end{matrix}$ | $\begin{matrix} u_{wall}=2.4\pi\sin\left(1.2\pi t\right)\text{ } \\ \text{ (cm/s)} \end{matrix}$ | $\begin{matrix} u_{wall}=4\pi\sin\left(2\pi t\right)\text{ } \\ \text{ (cm/s)} \end{matrix}$ |
| 2.6 | 0.281 | 1.320 | 2.732 |
| 2.7 | 0.279 | 1.320 | 2.732 |
| 2.8 | 0.277 | 1.320 | 2.732 |
| 2.9 | 0.276 | 1.320 | 2.732 |
| 3.0 | 0.274 | 1.320 | 2.732 |
| 3.1 | 0.273 | 1.320 | 2.732 |
| 3.2 | 0.272 | 1.320 | 2.732 |

**Table S2. Average shear stress provided by oscillator in a sinusoidal cycle (dyne/cm^2^)**

| **Film thickness (mm)** | **0.2 Hz** | **0.6 Hz** | **1.0 Hz** |
| --- | --- | --- | --- |
|  | $\begin{matrix} u_{wall}=0.8\pi\sin\left(0.4\pi t\right)\text{ } \\ \text{ (cm/s)} \end{matrix}$ | $\begin{matrix} u_{wall}=2.4\pi\sin\left(1.2\pi t\right)\text{ } \\ \text{ (cm/s)} \end{matrix}$ | $\begin{matrix} u_{wall}=4\pi\sin\left(2\pi t\right)\text{ } \\ \text{ (cm/s)} \end{matrix}$ |
| 2.6 | 0.179 | 0.840 | 1.739 |
| 2.7 | 0.178 | 0.840 | 1.739 |
| 2.8 | 0.176 | 0.840 | 1.739 |
| 2.9 | 0.176 | 0.840 | 1.739 |
| 3.0 | 0.174 | 0.840 | 1.739 |
| 3.1 | 0.174 | 0.840 | 1.739 |
| 3.2 | 0.173 | 0.840 | 1.739 |


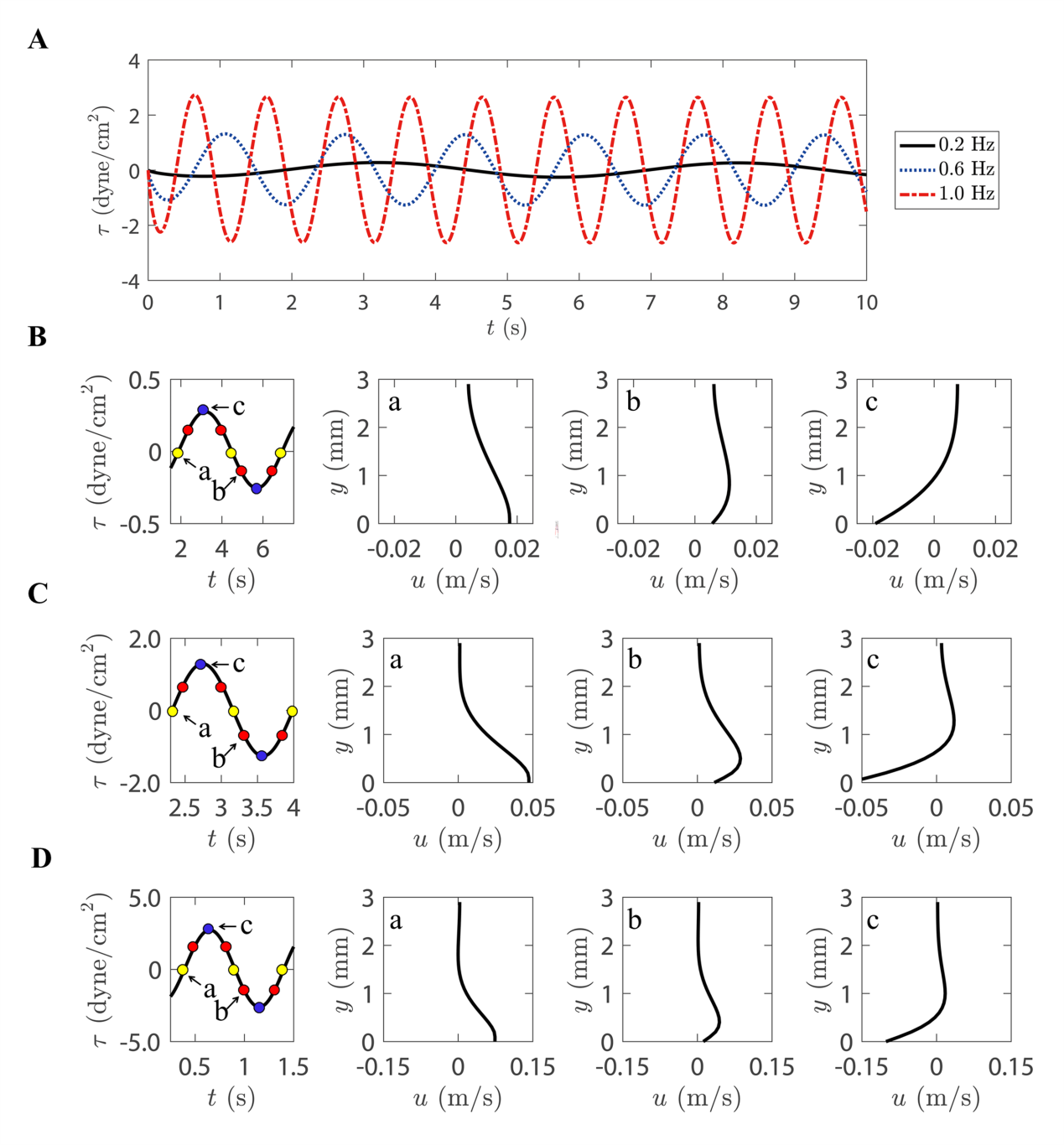


**Figure S3. The sinusoidal wave of shear stress and distribution of fluid velocity at 0.2 Hz, 0.6 Hz, and 1.0 Hz when the film thickness is 2.9 mm.** A, The sinusoidal wave of shear stress at different frequencies. B-D, The distribution of fluid velocity at different positions on the sinusoidal wave with the changes of y altitude under stresses of 0.2 Hz (B), 0.6 Hz (C), and 1.0 Hz (D). The plots of a, b, and c represent the distribution of flow rate at the minimum, intermediate, and maximum shear stresses, respectively. The points of a, indicated by yellow, representing the start, midpoint, and end of the curves; c, marked by blue, illustrating the peek and trough of the curves; b, noted by red, indicating the other intermediate points of the waves.

**
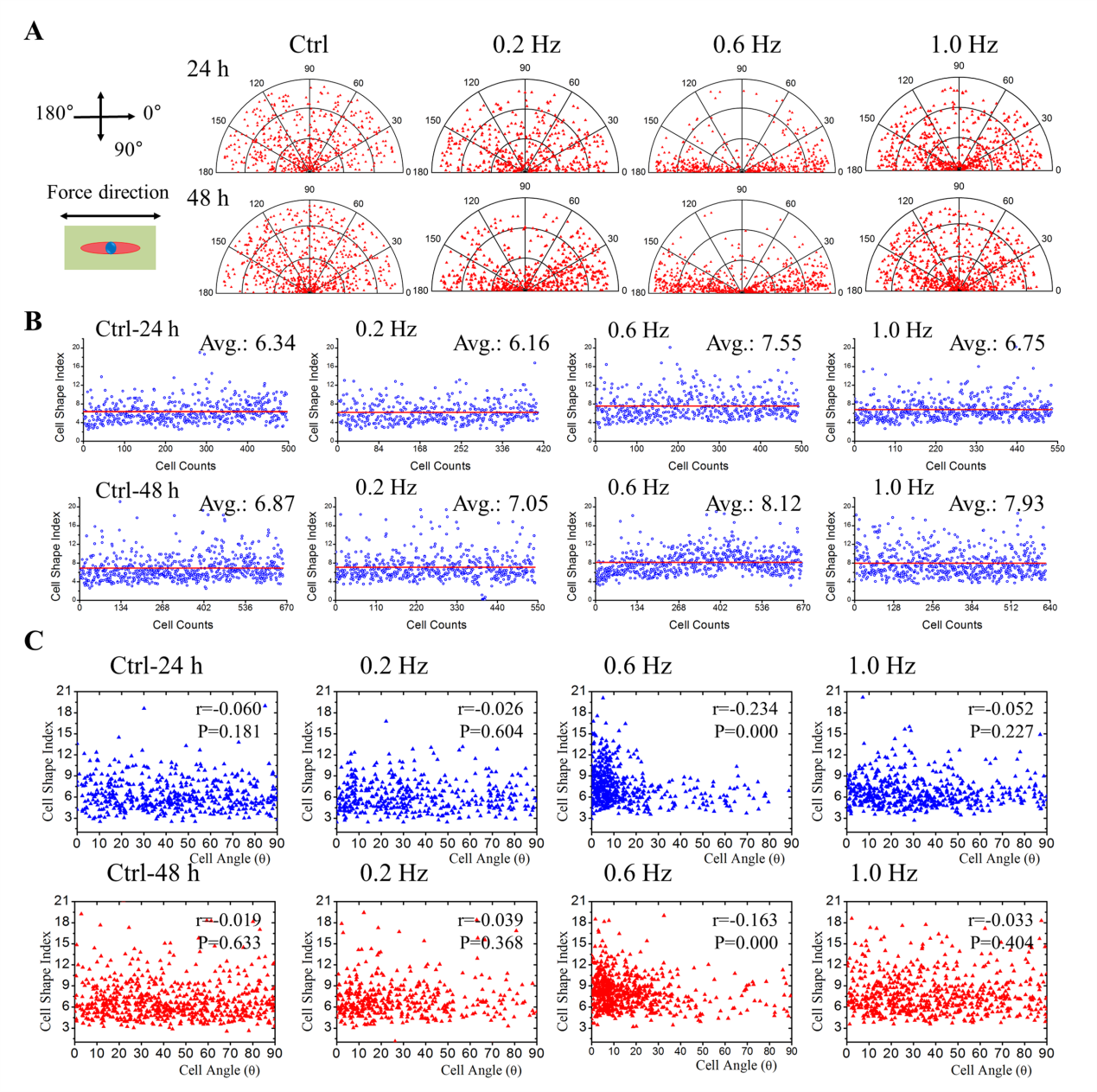
**

**Figure S4** **Morphological responses of NIH-3T3 cells implanted on plane to fluid stress for 24 or 48 h.** A, The cell orientation in the range of 0-180 degrees is shown with the polarity map. B, The scatter plot shows the CSI of fibroblasts. The red lines represent average value of CSI. C, Scatter plot of CSI versus cell angle. Each data point represents a single cell (A-C). Cell counts > 400 (A-C).

**
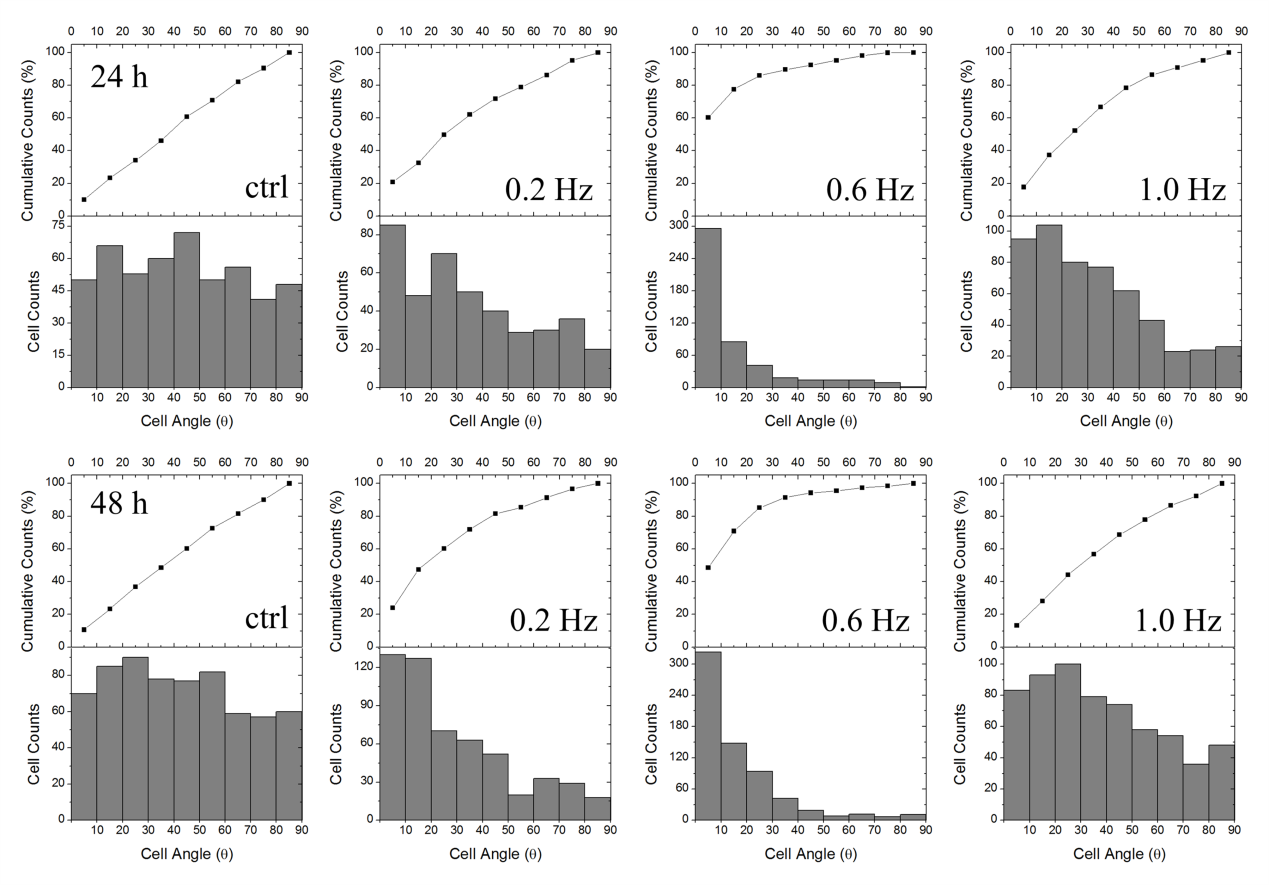
**

**Figure S5** **Cell angle distribution map.** The cell angle is evenly divided into 9 parts in the range of 0-90 degrees, quantifying the number of cells belonged to each part (lower) and the percentage of cumulative counts (upper).


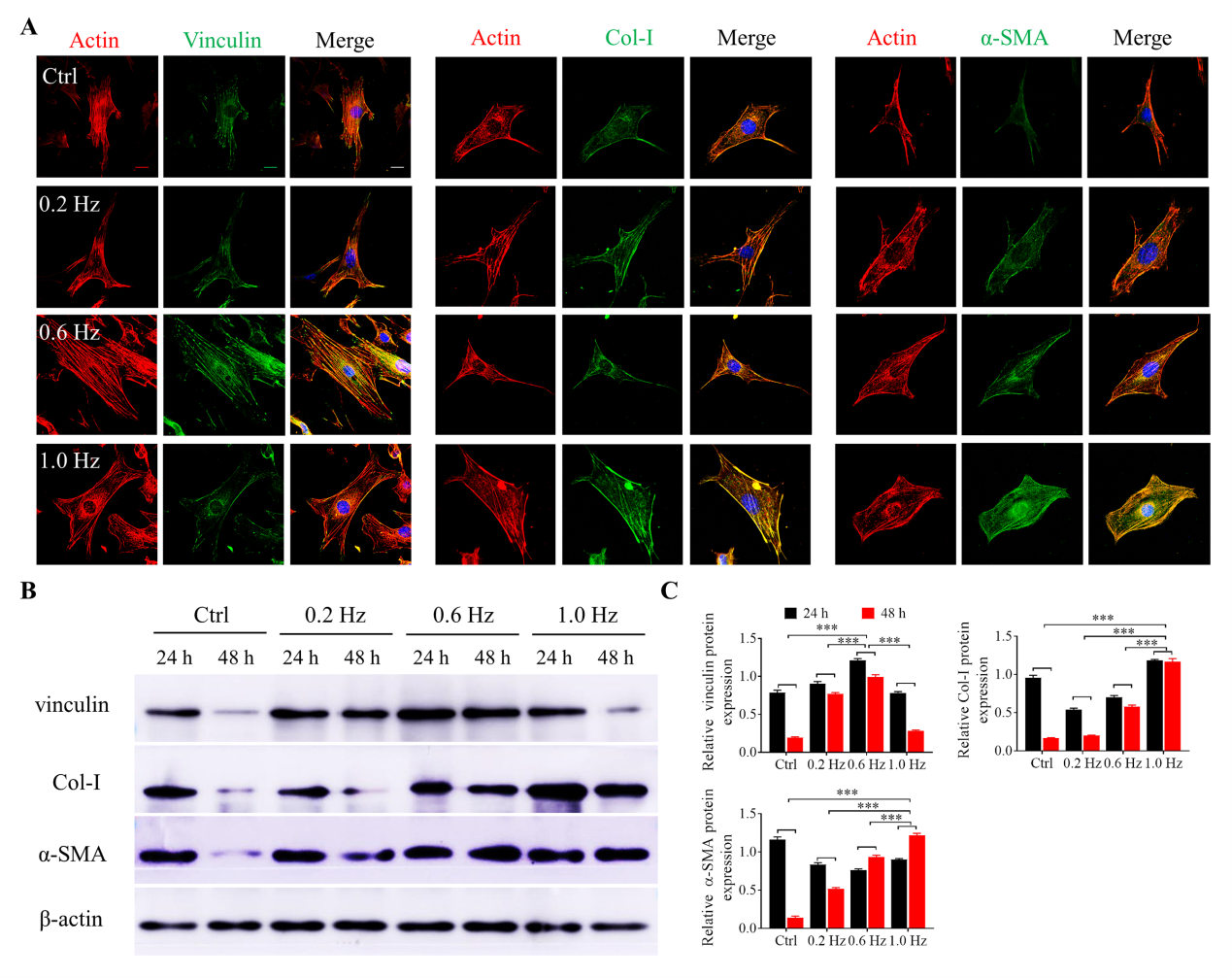


**Figure S6. Cell morphology and expression of vinculin (adhesion), Col-I and α-SMA (differentiation) proteins of NIH-3T3 cells incubated on plane substrates.** A, Cell morphology at 48 h under high power microscopy. Scale bars=20 μm. B, The expression levels of vinculin, Col-I and α-SMA are determined by Western blotting at 24 h and 48 h. C, Quantifying the gray value of protein bands, the proteins are normalized to β-actin, which served as an internal control. Two-way classification ANOVA, LSD, n = 3. Mean ± SD (C).

**
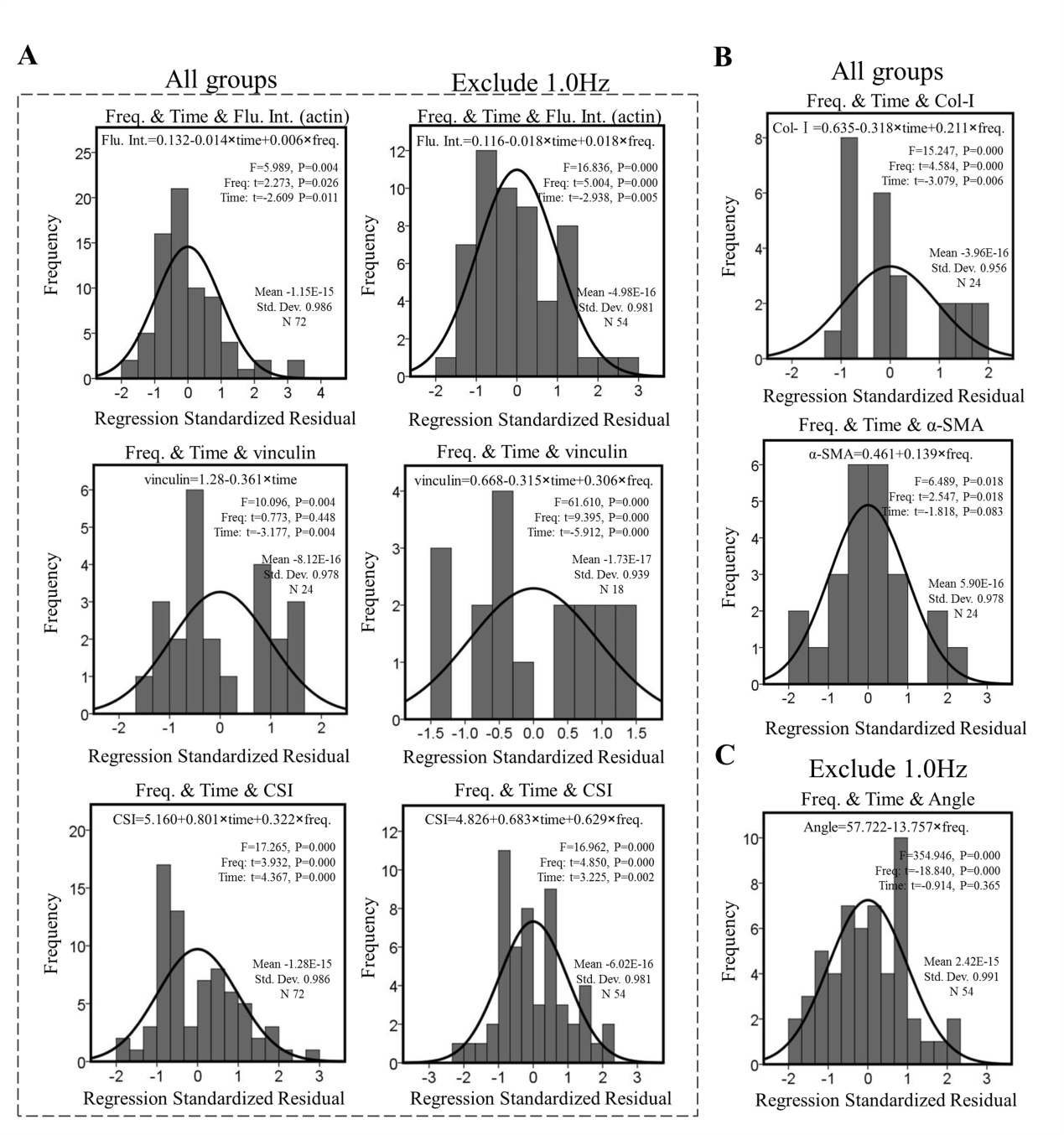
**

**Figure S7. Correlation analysis and schematic diagram of the effect of frequency on NIH-3T3 cells.** A, Histogram of all four groups versus other groups excluding 1.0 Hz from the multiple stepwise regression analysis on the correlation between frequency, time (as entered variables) and red actin fluorescence intensity, vinculin protein or cell shape index (as dependent variable). B-C, the correlation analysis between frequency, time and Col-I, α-SMA protein in all groups (B) or cell angle in groups excluding 1.0 Hz (C). The figure shows that actin fluorescence intensity, vinculin protein expression and CSI were more significantly correlated with frequency than time in all groups excluding 1.0 Hz. In addition, the frequency was more correlated with protein expression of α-SMA and Col-I than time in all groups.

**
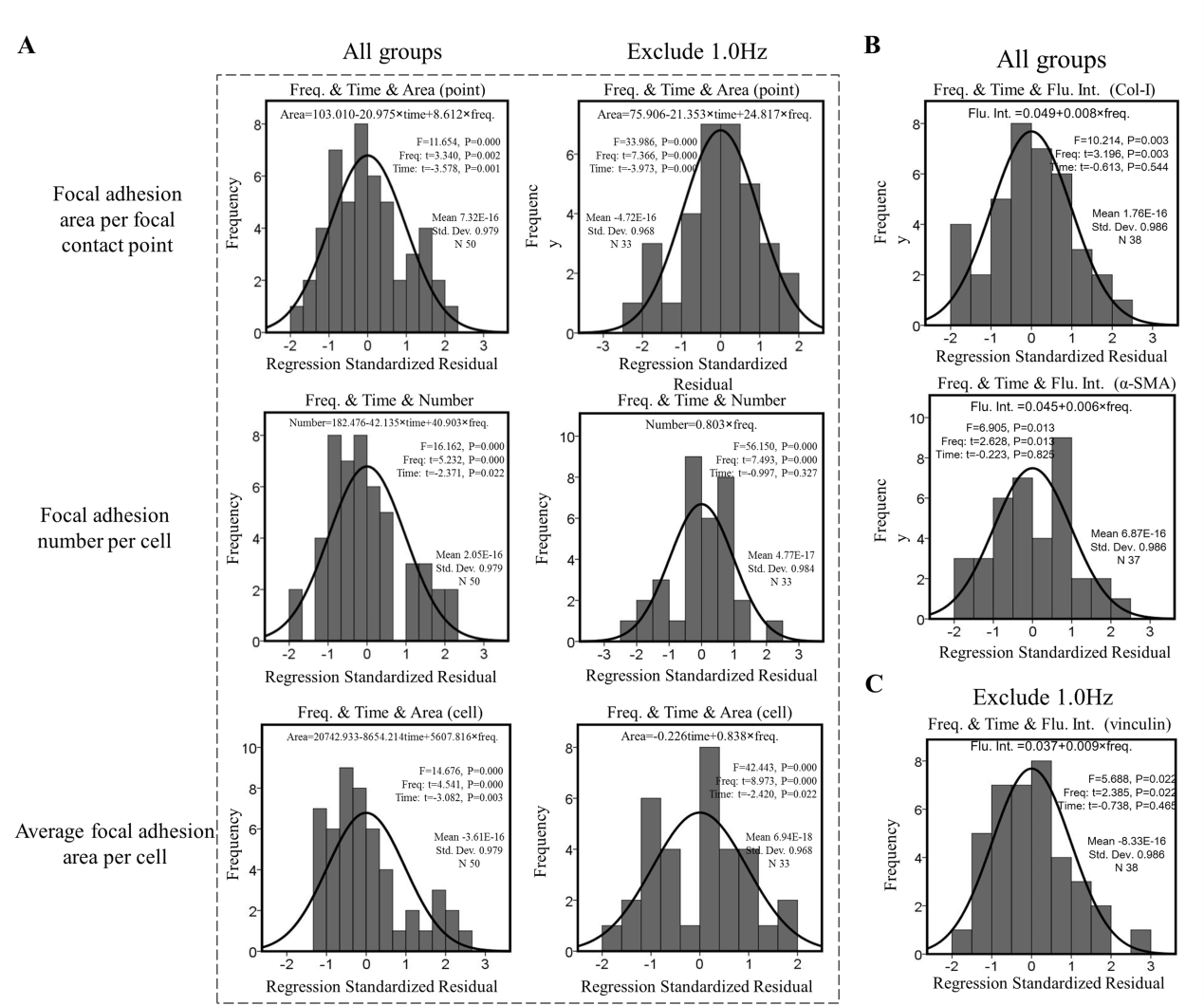
**

**Figure S8 Correlation analysis of the effect of frequency on NIH-3T3 cells.** A, Histogram of all four groups versus other groups excluding 1.0 Hz from the multiple stepwise regression analysis on the correlation between frequency, time (as entered variables) and focal adhesion area per focal contact point, focal adhesion number per cell or average focal adhesion area per cell (as dependent variable). Similarly, the correlation between frequency, time and fluorescence intensity of Col-I, α-SMA in all groups (B) or vinculin in groups excluding 1.0 Hz (C) were analyzed by the same statistical method. The figure shows the vinculin size, number and fluorescence intensity are more correlated with frequency after excluding the 1.0 Hz grouping factor (|t|_All groups_<|t|_Exclude 1.0 Hz_), compared with time, as well as cell angle. On the other hand, the frequency was more correlated with fluorescence intensity of α-SMA and Col-I than time in all groups.

**
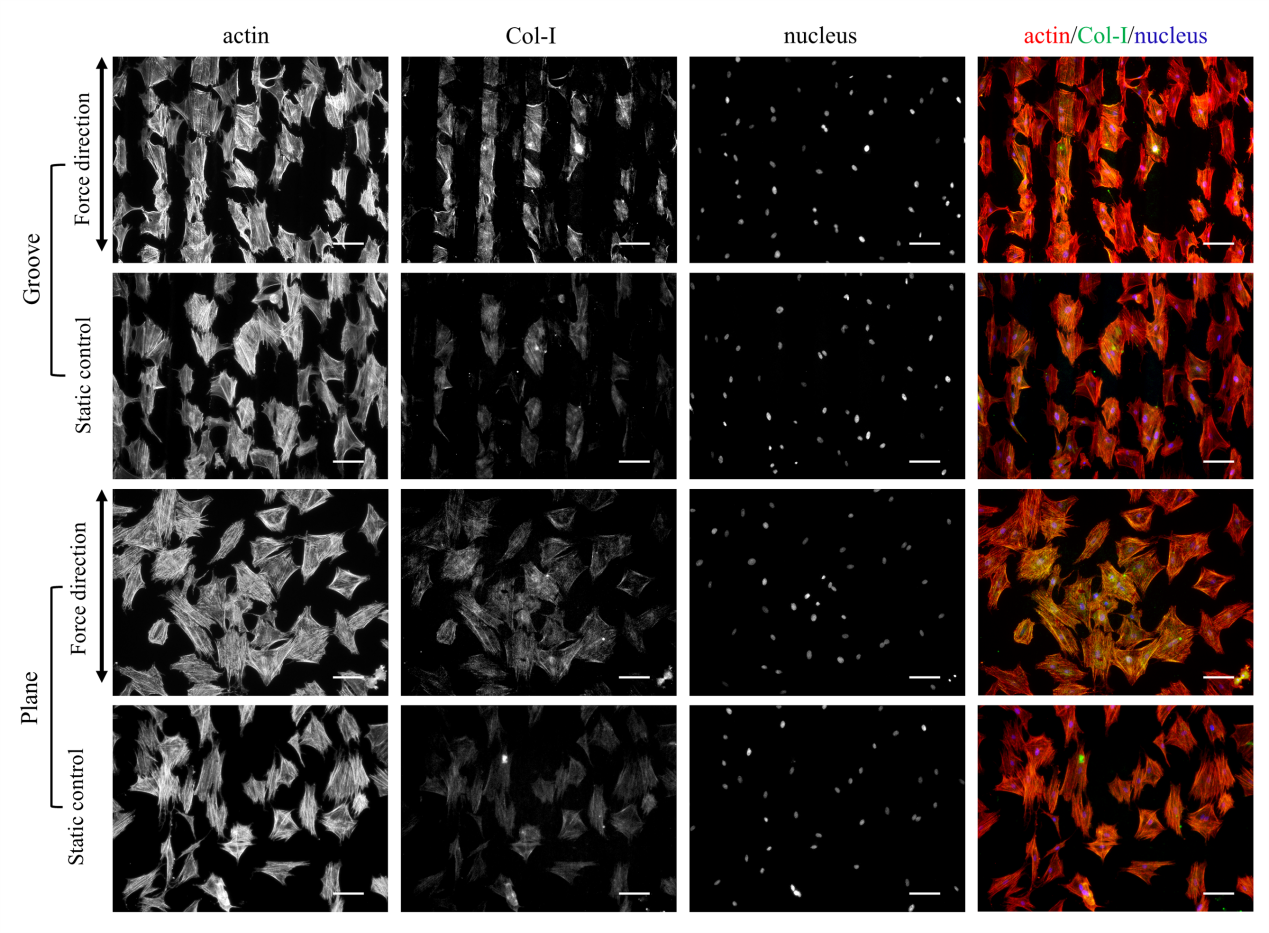
**

**Figure S9. Fluorescent images of cytoskeleton and Col-I of BMSCs seeded on plane and groove structures for 24 h.** Images are taken in black-and-white mode of fluorescence microscopy, and merged pictures are added with pseudo-color, including red (F-actin), green (Col-I) and blue (nucleus). The force direction is vertical, the static culture as a control. Scale bars: 100 μm.

[1] Li D, Cheng P, Jiang H, Cao T, Wang J, Gao Y, Lin Y, Wang C, Zhang S, Li J, Liu B, Song Y, Yang L, Pei G. Vascularization converts the lineage fate of bone mesenchymal stem cells to endothelial cells in tissue-engineered bone grafts by modulating FGF2-RhoA/ROCK signaling. Cell Death Dis. 2018; 9(10):959.
